# Proteome of the Luminal Surface of the Blood–Brain Barrier

**DOI:** 10.3390/proteomes9040045

**Published:** 2021-11-10

**Authors:** Jennifer J. Hill, Arsalan S. Haqqani, Danica B. Stanimirovic

**Affiliations:** Human Health Therapeutics Research Centre, National Research Council of Canada, Ottawa, ON K1A 0R6, Canada; Jennifer.Hill@nrc-cnrc.gc.ca (J.J.H.); Arsalan.Haqqani@nrc-cnrc.gc.ca (A.S.H.)

**Keywords:** blood–brain barrier, luminal, proteomics, brain endothelial cells, receptor-mediated transcytosis

## Abstract

Interrogation of the molecular makeup of the blood–brain barrier (BBB) using proteomic techniques has contributed to the cataloguing and functional understanding of the proteins uniquely organized at this specialized interface. The majority of proteomic studies have focused on cellular components of the BBB, including cultured brain endothelial cells (BEC). Detailed proteome mapping of polarized BEC membranes and their intracellular endosomal compartments has led to an improved understanding of the processes leading to internalization and transport of various classes of molecules across the BBB. Quantitative proteomic methods have further enabled absolute and comparative quantification of key BBB transporters and receptors in isolated BEC and microvessels from various species. However, translational studies further require in vivo/in situ analyses of the proteins exposed on the luminal surface of BEC in vessels under various disease and treatment conditions. In vivo proteomics approaches, both profiling and quantitative, usually rely on ‘capturing’ luminally-exposed proteins after perfusion with chemical labeling reagents, followed by analysis with various mass spectrometry-based approaches. This manuscript reviews recent advances in proteomic analyses of luminal membranes of BEC in vitro and in vivo and their applications in translational studies focused on developing novel delivery methods across the BBB.

## 1. Introduction

The neurovascular unit is an anatomically and functionally-defined entity that integrates brain vasculature and neuronal coupling. One of the distinct roles of this unit is to maintain brain homeostasis via exchange of nutrients across the brain endothelial cells (BEC) that form the blood–brain barrier (BBB) [1]. BEC form a physical barrier for hydrophilic compounds larger than 500 Da, due to the elaborate tight junctions between individual cells; they control bi-directional passage of nutrients, metabolic products, and large molecules via a rich complement of different classes of transporters, by virtue of phenotypic specialization and strict polarization. BEC specialization and function is influenced by the local brain microenvironment and the close proximity of other cellular and acellular components of the neurovascular unit, most notably pericytes, astrocytic end feet, perivascular macrophages, and neuronal endings.

Interest in the molecular makeup of the BBB stems from the need to understand its physiological functions and pathology and to devise strategies for improved delivery of therapeutics. The rise of genomics technologies in the 1990′s and 2000′s led to the first attempts to profile BBB mRNA expression levels using serial analysis of gene expression (SAGE) [2] and differential transcriptomic methods [3], mainly conducted using cultured BEC. Gel-based [4] and isotope-coded affinity tag (ICAT)-based [5] proteomic approaches were close to follow, contributing to improved understanding of how BEC react to inflammatory and hypoxic conditions. As genomics technologies advanced, the primary and cell lines generated from BECs, as well as emerging induced pluripotent stem cell (iPSC)-derived BEC-like endothelial cells, underwent extensive sequencing and cataloguing of their genotypes, enabling in-depth comparisons with peripheral endothelial and epithelial cell cultures [6,7,8]. Analyses of BEC outside of their natural microenvironment, proximity to interacting cells of the neurovascular unit, and de-differentiation in culture were the key drawbacks of these studies. In contrast, microarray-based differential transcriptomics in laser-capture micro-dissected brain microvessels [9,10,11] and RNA-Seq analyses of freshly isolated brain microvessels [12] captured more realistic in vivo expressed transcriptomes, with the caveat of ‘contaminating cells’ of the neurovascular unit always being present in the brain microvessels. Recently, single cell sequencing has been able to resolve these issues, enabling selective transcriptomic analyses of each different cell type within the neurovascular unit in brain tissues and vascular segments from in vivo samples, both mouse [13,14,15] and human [16,17]. This was a significant achievement and enabled BBB researchers to critically analyze and curate the extensive previous transcriptomics data sets from in vitro BBB models and isolated brain microvessels.

Ultimately, the field is seeking to complement these achievements with expressed proteomes with similar granularity, an even more challenging task. While transcript levels show some positive correlations with protein expression levels, protein expression is also modulated by several additional factors, including translational control, proteolysis, and cellular trafficking [18]. In addition, proteomic analyses can provide the required data on the proteins expressed in particular cellular locations, such as the cell surface. Advances in mass spectrometry (MS) instrumentation, methods, and data analysis algorithms continue to increase the utility of proteomic profiling experiments in the BBB field [19,20,21]. While the sensitivity of MS instrumentation has reached the levels required to analyze whole proteomes in small samples, even single cells, the challenges to the BBB field remain similar as for transcriptomics: the ability to separate specific cell types of an intricately intermingled neurovascular unit from fresh brain samples, as well as the ability to interrogate specific surfaces and intracellular compartments of BEC. Some persisting challenges the BBB poses to proteome analyses, include difficult-to-solubilize tight junction proteins and heavily post-translationally modified proteins, in particular through extensive glycosylation.

Mapping the expressed proteomes of the luminal BBB surface in vivo would be of pointed value for identifying highly-expressed endothelial proteins and transporters that could be exploited for brain drug delivery. Specifically, profiling luminal BBB proteins will be particularly useful to understand differences in protein expression between different species [22,23,24,25] and in various neurological diseases [26,27,28], which are both important considerations for the development of therapeutic delivery platforms.

**Figure 1:**
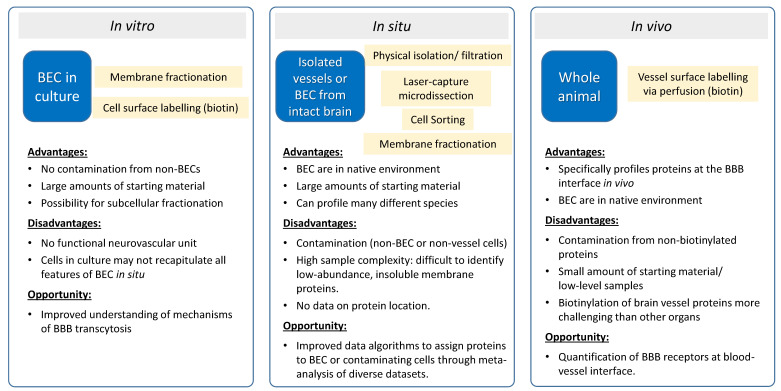
Model systems to study protein expression at the BBB.

This review surveys the progress in mapping proteomes of BEC with advanced MS technologies and with a particular focus on the most challenging frontier in this endeavor: surface-specific proteomics of the polarized BBB in vitro, in situ, and in vivo, as outlined in Figure 1.

## 2. Proteomics of In Vitro BBB Models

In vitro BBB models consist of BEC and are widely implemented in the (bio)pharma industry to aid in the pre-clinical evaluation and selection of prospective CNS-targeting pipelines [29]. They are also used to discover, localize, and validate ‘accessible’ membrane proteins in BEC as potential targets for therapeutics and brain delivery. In vitro BBB models provide several advantages over in vivo evaluation. Unlike in vivo studies, results generated from in vitro BBB models can specifically be attributed to the BEC, since they are free of contamination from other cell types of the neurovascular unit (e.g., astrocyte end-feet, pericytes). In addition, in vitro models allow the use of specialized techniques that would be difficult to implement in vivo, such as allowing enough materials for membrane isolation methods for proteomics analysis (see Figure 1).

Several studies have coupled cell-surface capturing methods with proteomics and targeted MS methods to discover and localize ‘accessible’ proteins in various membranes of BBB models. Ito et al., (2020) [30] used a biotin-labeling methodology to capture membrane proteins with accessible amines sites on the surface of a human BBB model consisting of immortalized human brain endothelial hCMEC/D3 cells and carried out proteomics using an advanced quantitative MS technique (SWATH-MS, a data-independent acquisition method) to identify 125 cell-surface proteins. They were successful in detecting key receptors that undergo receptor-mediated transcytosis (RMT), including TFRC, LDLR, and IGF1R, on the apical (luminal) surface, demonstrating the suitability of these cells for transcytosis evaluation. In addition, they identified several additional receptors as potentially novel RMT-based brain delivery targets. However, several of the prominent RMT receptors, such as SLC2A1 (GLUT1), LRP1, and INSR could not be detected, despite the fact that some of these proteins have several accessible amine sites and were detected in previous studies in the same cells [31]. Simonian et al., (2017) [32] also used a similar biotin-labeling technology, followed by proteomics and targeted MS analysis, to capture surface proteins on a mouse BBB model consisting of bEND.3 cells. They identified at least 50 apical membrane proteins, including several that showed differential expression in response to irradiation. While several endothelial cell-specific proteins were detectable, several BBB-specific receptors were still lacking in the proteomics results.

Density gradient fractionations have also been used to obtain purified preparations of apical, basolateral, and endosomal membranes from BBB. The strength of these techniques is that specific receptors of interest can then be examined using biochemical assays [33,34] or proteomics/MS analysis [35,36,37,38,39]. In a set of studies, we utilized density gradient fractionation to isolate apical, basolateral, endosomal, and exosomal proteins from immortalized BBB cells of either human (hCMEC/D3) or rat (SV-ARBEC) origin [35,36,37,38,39]. The fractions were analyzed by proteomics and targeted MS to localize and relatively quantify RMT receptors and ABC transporters. These methods allowed the identification of polarized levels of >150 receptors on the apical and basolateral membranes [35,36], including prominent RMT receptors such as TFRC, LDLR, IGF1R, SLC2A1, LRP1, and INSR, as well as potentially novel BBB receptors. In addition, they identified at least 14 ABC transporters with polarized levels on either the apical or basolateral membranes. These methods also allow simultaneous quantitative ‘tracking’ of the RMT receptors and BBB-crossing antibodies that target these receptors in various endosomal compartments [37,38]. The results from these studies have led to a better understanding of the potential mechanisms of BBB transcytosis by antibodies, including the potential role of exosomes [37,39] in the process. In addition, the methods facilitate the optimization of antibodies to improve the transcytosis of therapeutics, something that would be difficult to do in vivo.

## 3. Vessel Proteomics In Situ

As discussed above, proteomic studies of polarized endothelial cells in culture have provided rich data sets of cell surface proteins that are enriched on the luminal side of BEC. To better understand how protein expression may differ between in vitro models and vessels in situ, it is also important to profile luminally-exposed proteins in the context of a fully-functional vessel [40]. For example, over 40% of the proteins identified in the luminal membrane fraction of rat lung vessels were not identified in cultured rat lung endothelial cells [41]. While technical limitations may account for some of these ‘missing’ identifications, Western blot analysis in this same study confirmed that at least 14 known vascular endothelial proteins were found only in the isolated vessels and not in the cultured endothelial cells, suggesting that endothelial cells in culture do not fully recapitulate the endothelial cell surface in vivo. Isolated vessels and BEC from intact brain tissue may more fully reflect the situation in vivo, and various isolation and membrane-enrichment procedures have been explored for this purpose. While the proteomic profiling of isolated vessels and cells does not specifically provide information on the location of the proteins on the luminal vessel surface, these data sets are often rich in luminal proteins and can provide a valuable resource for the identification of luminally-expressed receptors, which are relevant to BBB drug delivery discovery efforts (see Figure 1).

### 3.1. Brain Vessel Isolation

Several groups have produced proteomics data that profile and quantify the proteins present in isolated brain vessels from rodents, monkey, and human [42]. Most of these studies were previously reviewed in detail in a 2015 review article [43]. Of these earlier studies, only one study specifically focused on membrane proteins, which are of particular interest as potential proteins that may be expressed on the luminal vessel surface. In this study, Chun et al. (2011) combined a well-validated vessel isolation and membrane enrichment strategy with a comprehensive multidimensional chromatography (Mud-PIT) based proteomic analysis, leading to the identification of 1143 proteins with high confidence, of which 50% were predicted to be associated with membranes [44]. A comparison of the protein identifications with a published list of predicted cell surface proteins [45] suggests that a significantly smaller proportion of proteins (14%) are likely to be expressed on the cell surface and, therefore, be possible candidates for transport of therapeutics through the BBB. Importantly, many known BBB RMT proteins were identified, suggesting very good coverage of vessel luminal proteins in this data set (see Table 1). In addition, several known markers of pericytes and astrocytes were also identified. More recently, Al Feteisi et al. (2018) developed an optimized brain vessel isolation and sample preparation procedure, which was validated through mRNA measurement of known markers for ECs, pericytes, neurons, and astrocytes [46]. Using this method, 1897 vessel-associated proteins were identified. Of these, 10% were plasma membrane proteins, including several known RMT proteins (Table 1). This data set was also used to estimate the abundance of 26 transporter, receptor, and marker proteins, relative to the expression of Atp1a1 and Abcb1, providing valuable data for PK/PD modeling at the BBB. A different method for isolating brain vasculature, surgical isolation of larger brain cerebral arteries, identified over 6000 proteins, including several known BBB RMT proteins (see Table 1), and ultimately led to the identification of 192 cerebrovascular proteins that were differentially-expressed in a mouse model of Alzheimer’s disease [28,47]. In 2020, Campeau et al. profiled changes in the BBB upon Group B *Streptococcus* infection using proteomic analysis of isolated vessels, identifying over 4700 proteins; the most comprehensive proteomic analysis of brain vessel proteins to date [48]. In this study, proteins from 3511 unique gene identifiers were found in all analyses, of which 10% were cell surface proteins, including most known RMT markers (see Table 1), suggesting a high depth of coverage.

### 3.2. Laser-Capture Microdissection of Brain Vessels

Earlier studies have isolated vessels from the brain using laser-capture dissection (LCM), and these have been reviewed previously [5,43,49]. One newer study by Zajec et al. built on this earlier work by using LCM, together with analysis on a next-generation Orbitrap tribrid mass spectrometer [50]. Using this approach, 1882 proteins were identified with at least two spectral counts in micro-dissected intracerebral microvessels, arachnoidal vessels, and brain tissue. Of these proteins, 8% were predicted cell surface proteins, highlighting the sample preparation challenge with small amounts of tissue isolated by LCM, where hydrophobic cell surface proteins are often lost during processing. In this study, the authors described a short list of 14 proteins that were exclusively identified in intracerebral microvessels, but not in arachnoidal vessels or surrounding tissue. Interestingly, five of these proteins are present on the cell surface and three are associated with the extracellular matrix, suggesting that cell surface proteins may be enriched in markers of BEC and vessels.

### 3.3. Isolated Brain Endothelial Cells

Proteomic studies on isolated vessels have identified a variety of known EC markers with luminal expression. However, while these data sets are typically highly enriched in endothelial cell proteins, these proteomic studies also identified proteins from pericytes, astrocytes, glial cells, and even neuronal cells, due to the complex nature of the blood vessel and imperfections in the vessel isolation procedure. In many cases, the identified proteins can be assigned to ECs through comparison to the growing number of single-cell RNA-Seq data sets from individual brain cells [13,14,15,16,17,51,52,53,54]; however, this analysis provides no information on the subcellular location of these proteins. Proteomics can also be used to directly interrogate protein expression in isolated endothelial cells from brain, as demonstrated by Zuchero et al. [55]. Following isolation of CD31-positive/CD45-negative endothelial cells from mouse brain by fluorescence-activated cell sorting (FACs), peptide counts with MS were used as a surrogate for abundance, to identify proteins that are much more highly expressed in BEC, relative to negatively selected non-BEC lysates. While the full proteomic data set was not disclosed, the authors identified several transmembrane proteins that showed BEC enrichment, including SLC2A1, TFRC, BSG, and SLC3A2.

## 4. Luminal Proteomics In Vivo

The identification and quantification of luminal proteins that are exposed to the blood stream is of particular interest in the development and evaluation of many types of therapeutics, including BBB delivery systems. Immunohistochemistry can potentially confirm a luminal localization for an individual protein; however, due to the narrow distance separating luminal and abluminal membranes in BEC, it can be technically challenging to distinguish luminal and abluminal locations using many microscopy approaches [56]. In addition, quantification of luminal expression by these methods is very challenging. For this reason, relatively little is known about the abundance and types of proteins that are specifically present on the luminal surface of brain vessels. When combined with specific membrane fractionation and data analysis methods, isolated vessels have proven useful for the identification and quantification of luminal membrane protein expression in brain microvessels. The majority of studies that specifically profiled and quantified proteins with luminal vessel expression used chemical labelling methods aimed at labelling blood-accessible proteins (see Figure 1). Proteomic analyses using both of these approaches are highlighted in the sections below.

### 4.1. Vessel Membrane Fractionation

One approach to deciphering the luminal versus abluminal expression of vessel proteins was demonstrated for a subset of preselected proteins-of-interest by Kubo et al. [57]. In this work, an extension of the vessel isolation procedure was developed that relied on further fractionation of vessel membranes within a sucrose/ficoll density gradient. Using a targeted MS method, each fraction was analyzed to quantify the abundance of ten vessel proteins, including a luminal marker (MDR1), an abluminal marker (ATA2), and a general vessel marker (the Na+/K+–ATPase). This experimental design allowed for correction of cross-contamination between fractions, leading to an estimation of the luminal and abluminal distribution ratio for each of the quantified transporters. This approach required a large amount of material due to the low yield of membrane fractions, and only a limited number of transporter proteins were quantified. With the recent improvements in MS instrument capabilities, it would be interesting to determine if an untargeted proteomics approach might be able to quantify the relative luminal and abluminal expression using this same fractionation approach, combined with a label-free or isobaric label-based quantification method; however, to our knowledge this possibility has not yet been explored.

### 4.2. Perfusion-Based Chemical Labelling Methods

The vast majority of proteomic studies, focused on luminal protein expression in vessels, have relied on terminal perfusion techniques to chemically modify proteins that line the vessel surface. Terminal perfusion is frequently used in animal studies to remove the blood from tissues or to add fixative for staining studies [58]. For luminal protein profiling experiments, it is important to optimize the perfusion technique, to ensure that the pressure during perfusion is steady and does not rupture the microcapillaries, which would lead to leakage of the labelling agent and subsequent labelling of non-vessel proteins. For this reason, it is ideal to use a pump system to allow a steady perfusion with consistent pressure throughout the experiment.

One of the earliest of these perfusion-based methods utilized colloidal silica and a crosslinker to coat the inside of the vessel, allowing the isolation of luminal membranes with high enrichment [59]. Since the entire luminal membrane is enriched in this method, these data sets include a high proportion of cytoskeletal and inner peripheral membrane proteins that are not necessarily accessible to the blood. This silica-coating method was successfully applied to a proteomic study on rat lung vessels in 2009. In an extensive study covering many of the major proteomic methods used at the time, the authors were able to identify 1800 proteins, with ~25% representing transmembrane proteins [60]. Although this isolation method appears interesting, to our knowledge, the protein profiling data from brain vessels were never published; although this analysis was alluded to in a 2004 publication, where a 2D-gel image of brain luminal membrane proteins was shown [61].

Terminal perfusion has most commonly been used to specifically label blood-accessible proteins through chemical modification using an N-hydroxysuccinimide (NHS)-ester activated biotin. Biotin covalently labels proteins on the surface of the vessel via exposed primary amines on lysine residues or the N-termini of proteins, which are then enriched using streptavidin followed by MS analysis. This method was first utilized for a proteomics study in 2005 by Rybak et al., who used sulfo-NHS-LC-biotin to label blood-accessible proteins in kidney, liver, muscle, and heart, as well as two experimental tumor models, identifying between 44 and 155 proteins in each tissue [62]. Of these proteins, between 5–24% were membrane proteins, with an additional 1–29% of the proteins representing extracellular matrix proteins, depending on the tissue. The relatively high proportion of intracellular proteins identified suggested a lack of specificity in this method, perhaps due to some non-specific binding during the streptavidin isolation, although the authors speculate that some of the biotin reagent may have also crossed the cellular membrane. A large number of blood proteins were also identified, especially in muscle and tumor samples, suggesting that some of these tissues may be difficult to effectively perfuse, and highlighting one of the challenges in perfusion-based luminal labelling methods. In 2008, Roberts et al. [56] used perfusion-based biotinylation, followed by immunofluorescence and Western blot analysis of biotinylated and non-biotinylated fractions, to localize 11 transporters that had been previously identified as likely endothelial cell markers from a qPCR analysis of enzymatically isolated rat BEC. These targeted methods confirmed the luminal expression of several known and suspected BBB transporters.

In a proteomics context, numerous studies have isolated luminal vessel proteins from non-CNS tissues such as subcutaneous tumors, liver, and kidney, following in vivo biotinylation [62,63,64,65,66,67,68,69]. However, we are only aware of two publications that provided proteomic profiling of proteins isolated from brain vessels using this method. In 2011, Roesli et al. utilized NHS-biotin perfusion in a transgenic mouse model of beta-amyloidosis to identify accessible vascular proteins in the cortex [70]. Through this workflow, 177 proteins were identified, of which 25% were predicted to be cell surface. These cell surface receptors included several known RMT receptors, such as IGF1R, SLC2A1 (Glut1), and SLC3A2 (CD98 hc) (see Table 1). Interestingly, the authors noted that the number of proteins identified from the brain vasculature was approximately five-fold lower compared to similar analysis in other mouse organs, suggesting that biotin labelling in brain vessels may be more difficult than in other organs. Interestingly, the same result was seen in a more recent perfusion-based labelling experiment that was performed by Toledo et al., using a new generation Orbitrap-based mass spectrometer [69]. This study was focused on the identification of vascular markers for sepsis, with an emphasis on liver and kidney vessels. However, proteomic profiling data for biotinylated proteins were also provided from brain, heart, and white adipose tissue. Interestingly, only 96 proteins were identified in brain vessels, compared to the 317, 319, 289, and 242 proteins identified in liver, kidney, heart, and white adipose tissue, respectively. This finding appears to confirm that biotin-labelling and isolation of proteins from brain vessels is more challenging than from many other tissues. Furthermore, of the 96 proteins identified in brain vessels, only 12 were predicted to be cell surface proteins (Table 1) and none of these represented known RMT receptors, suggesting that a low depth of coverage and a lack of specificity for these biotin-based methods continues to be an issue.

In an unpublished work, our group has developed a perfusion-based approach using an alternative labelling approach, which enables highly-specific isolation of surface-exposed proteins from brain vessels. This approach also implemented a vessel enrichment step to improve protein recoveries and minimize non-specific protein identifications. In preliminary applications of this method, in both rat and mouse, 193 accessible vessel proteins were identified, of which 75% were cell surface proteins (Table 1), suggesting that this method may provide higher specificity for luminal vessel proteins than the published biotinylated studies described above. Importantly, a high percentage of known RMT receptors were identified in these studies, including INSR, IGF1R, TFRC, SLC2A1, LRP1, SLC3A2, and BSG (Table 1), suggesting that this method has promise for more complete profiling of the luminal proteome in the future.

**Figure 2:**
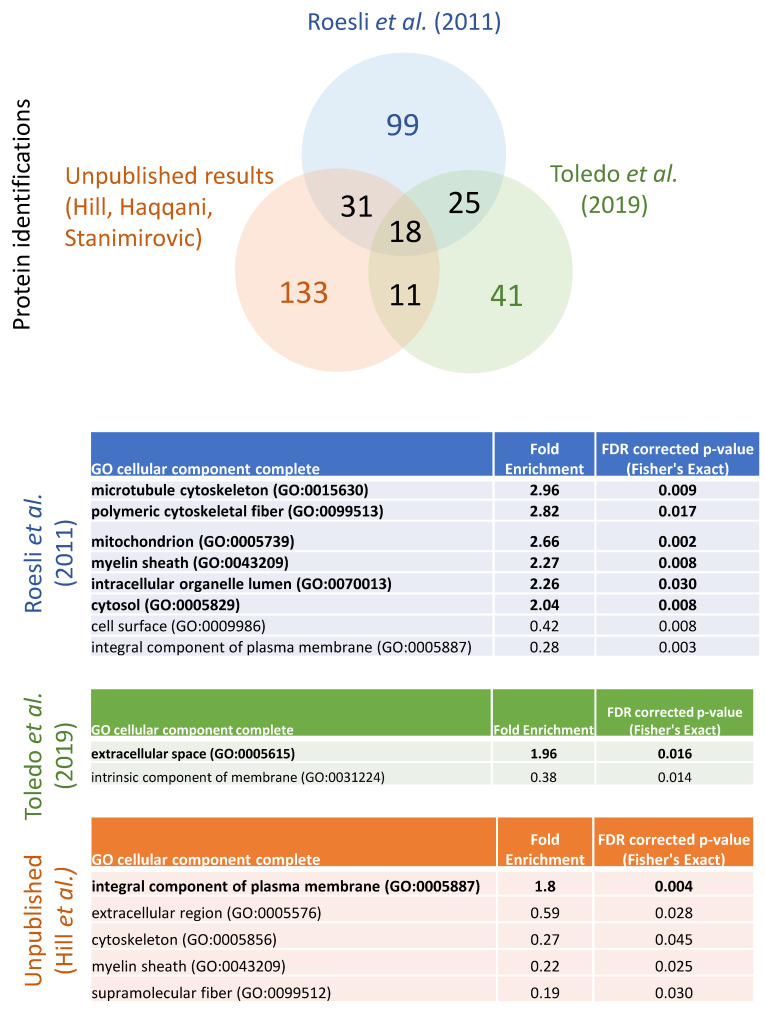
Comparison of in vivo luminal proteomic studies in brain using perfusion-based chemical labelling. Top: Venn diagram depicting the overlap in protein identifications in three in vivo luminal proteomic studies in brain, Roesli et al. [70], Toledo et al. [69] and unpublished results from our group. Bottom: Panther statistical overrepresentation analysis on GO cellular component terms for the unique set of proteins identified in each of the three studies, using a reference list composed of all proteins identified in the three experiments. Overrepresented categories are in bold text.

A comparison between the two published brain luminal proteomic data sets and our unpublished results shows a relatively low level of overlap in the protein identifications between the three data sets (Figure 2). In total only 18 proteins were identified in all three experiments, and of these only six were predicted to be cell surface proteins: ALPL, BCAM, CDH5, ITGB1, PODXL, and SEMA7A. Conversely, over 75% of the identified proteins were only identified in one of the three experiments. To determine the types of proteins that were identified only in one data set, a statistical overrepresentation analysis was run using Panther [71]. The only gene ontology (GO) category showing statistically significant enrichment after FDR correction was cellular component, which reveals that Roesli et al. [70] identified an overrepresentation of cytoskeletal, mitochondrial, myelin sheath, intracellular organelle lumen, and cytosolic proteins relative to the proteins identified in all three studies. Some of these differences may reflect specific proteins that are relevant to the beta-amyloidosis mouse model that was included in this study, although these classes of proteins were not generally identified as overexpressed in beta-amyloidosis in their published analysis. Brain luminal proteins that were identified by Toledo et al. [69] were overrepresented by extracellular space proteins, perhaps due to challenges in getting complete perfusion of brain leading to blood contamination. Our unpublished results show an enrichment in integral plasma membrane proteins, relative to the other two studies, suggesting that our method may enable a higher level of labelling specificity for surface proteins.

## 5. Beyond Proteomic Profiling for In Vivo Luminal Protein Profiling in Vessels: Alternative Methods to Identify Potential Luminal RMT Targets In Vivo

Large-scale efforts to characterize the human proteome have been put to good use by researchers in the BBB field. The Human Protein Atlas has provided a publicly-accessible immunohistochemical analysis for more than 20,000 human proteins across a variety of normal tissues, including the brain [72]. This data was mined in a comprehensive effort by Lee et al., who highlighted a subset of 346 proteins that show expression in blood vessels in the brain cortex and then further categorized these proteins based on their expression in ECs and vascular smooth muscle cells [73]. By comparison with other human tissues, eight proteins were flagged as being specific to BECs; interestingly, two of these proteins are known RMT proteins, SLC2A1 and SLC3A2. While this analysis did not specifically identify luminal proteins, it provides important context for the further analysis of luminal-focused proteomic data sets.

The advent of accessible single-cell RNA-Seq (scRNA-Seq) technologies has enabled the direct correlation of a cellular phenotype with the expression level of individual genes. One interesting recent study, relevant to the BBB, highlighted the potential of these ‘indirect’ approaches to identifying luminal proteins responsible for the internalization of plasma proteins into BEC in vivo [74]. Following treatment of mice with fluorescently-labelled plasma proteins, 745 individual BEC were analyzed by scRNA-Seq. Next, genes whose expression levels were positively correlated with the amount of fluorescently labelled plasma proteins internalized in each of the 745 BEC were identified. Intriguingly, the top 1% of positively correlated genes (consisting of 199 genes) included >40 (20%) cell surface proteins, and at least six of these were known RMT proteins (INSR, IGF1R, TFRC, SLC2A1, SLC3A2, and BSG). As these new types of technologies continue to develop, they will almost certainly play a critical role in identifying new functional transporter proteins at the BBB.

## 6. Conclusions

To date, many proteomic data sets describing protein expression in endothelial cells and vessels have been published, but relatively few of these proteomic studies were designed to specifically identify and quantify brain luminal vessel proteins. Cell surface proteomics and density gradient fractionation of polarized ECs in culture have provided numerous data sets and candidate protein targets for BBB therapy delivery, with a high depth of coverage. In vivo, most known RMT receptors at the BBB have been identified only in proteomic studies of isolated vessels, providing little data on the proportion of these protein molecules that sit at the luminal membrane. Perfusion-based chemical labelling approaches, which specifically label and capture accessible luminal proteins in vivo, are a promising approach to profile the luminal proteome at the BBB. However, the limited data available suggests that brain vessel surface proteins may be intrinsically challenging to chemically label. Improvements in mass spectrometer instrument sensitivity may improve these results, as it has been estimated that there is only 1 µL of brain capillary endothelial intracellular volume in an entire rat brain [75]. In addition, it is possible that inefficient perfusion of the capillaries in the brain using transcardial perfusion and/or the unique glycocalyx at the BBB may limit the effectiveness of chemical labelling techniques. Improvement to the perfusion methods, combined with adaptation of glycosylation labelling approaches, such as metabolic labelling of azide-containing sugar molecules in the glycocalyx [76], may provide opportunities to improve the specificity and depth of coverage of luminal protein profiling. As methods develop and improve, we expect that a wider variety of RMT receptors will be identified in the brain, leading directly to the development of improved BBB carrier molecules. The ability to identify and quantify internalization of cell surface receptors from the vessel surface in vivo would be of particular interest for the BBB transport field, perhaps through the adaptation of internalization proteomic methods that have been applied to cells in vitro [30,37,38]. As methods and technologies improve, the quantification of the luminal expression levels of proteins at the BBB will enable an improved understanding of the features of RMT-mediated transport of the BBB, leading to many new opportunities to effectively treat and cure CNS diseases.

## Figures and Tables

**Table 1:** BBB RMT transporters identified in vessel proteomic studies.

Study	Sample	# Unique Proteins Identified	% Cell Surface Proteins ^a^	RMT Receptors (HGNC Symbol) ^b^											
				INSR	IGF1R	TFRC	SLC2A1	LRP1	TMEM30A	SLC3A2	FCGRT	BSG	LEPR	LRP8	LDLR
Proteomics of isolated vessels * (membrane focus or post-2015)															
Chun et al., (2011)	enriched mouse brain vessels followed by membrane isolation	1143	14%	?	Y	Y	Y	Y	?	Y	Y	Y	N	N	N
Badhwar et al., (2014, 2017)	surgical enrichment of mouse brain arteries	6630	11%	?	N	N	Y	?	?	Y	?	N	N	N	N
Al Feteisi et al., (2018)	enriched rat brain vessels	1897 ^c^	10%	N	N	Y	Y	Y	Y	Y	Y	Y	N	N	N
Campeau et al., (2020)	enriched mouse brain vessels	3511 ^c,d^	10%	Y	Y	Y	Y	Y	N	Y	Y	Y	N	N	?
Zajec et al., (2021)	LCM of human brain vessels	1882	8%	N	N	N	Y	Y	N	Y	N	Y	N	N	N
Proteomics of perfusion labelled blood-accessible vessel proteins															
Roesli et al., (2011)	perfusion of sulfo-NHS-LC-biotin; enrichment of labelled proteins from mouse brain	177	25%	N	Y	N	Y	N	N	Y	N	Y	N	N	N
Toledo et al., (2019)	perfusion of sulfo-NHS-biotin; enrichment of labelled proteins from mouse brain	96	13%	N	N	N	N	N	N	N	N	N	N	N	N
Unpublished results (JJH, ASH, DBS-2021)	perfusion-based labelling approach, vessel enrichment, and enrichment of labelled proteins from mouse and rat brain	193	75%	Y	Y	Y	Y	Y	N	Y	N	Y	N	N	N
Alternative ‘omics approaches															
Yang et al., (2020)	Top 1% genes showing positive correlation between scRNAseq expression and amount of plasma protein uptake in brain ECs	199	20%	Y	Y	Y	Y	N	N	Y	N	Y	N	Y	N

^a^: based on comparison to Bausch-Fluck et al. [45]; ^b^: Y = protein identified; N = protein not identified; ? = protein identified with low confidence; ^c^: duplicate gene symbols removed; ^d^: ‘common’ list of more confident identifications.
